# CellVisioner: A Generalizable Cell Virtual Staining Toolbox based on Few-Shot Transfer Learning for Mechanobiological Analysis

**DOI:** 10.34133/research.0285

**Published:** 2023-12-05

**Authors:** Xiayu Xu, Zhanfeng Xiao, Fan Zhang, Changxiang Wang, Bo Wei, Yaohui Wang, Bo Cheng, Yuanbo Jia, Yuan Li, Bin Li, Hui Guo, Feng Xu

**Affiliations:** ^1^ The Key Laboratory of Biomedical Information Engineering of Ministry of Education, Xi’an Jiaotong University, Xi’an 710049, P.R. China.; ^2^ Bioinspired Engineering and Biomechanics Center (BEBC), Xi’an Jiaotong University, Xi’an 710049, P.R. China.; ^3^ Department of Medical Oncology, The First Affiliated Hospital of Xi’an Jiaotong University, Xi’an 710061, P.R. China.

## Abstract

Visualizing cellular structures especially the cytoskeleton and the nucleus is crucial for understanding mechanobiology, but traditional fluorescence staining has inherent limitations such as phototoxicity and photobleaching. Virtual staining techniques provide an alternative approach to addressing these issues but often require substantial amount of user training data. In this study, we develop a generalizable cell virtual staining toolbox (termed CellVisioner) based on few-shot transfer learning that requires substantially reduced user training data. CellVisioner can virtually stain F-actin and nuclei for various types of cells and extract single-cell parameters relevant to mechanobiology research. Taking the label-free single-cell images as input, CellVisioner can predict cell mechanobiological status (e.g., Yes-associated protein nuclear/cytoplasmic ratio) and perform long-term monitoring for living cells. We envision that CellVisioner would be a powerful tool to facilitate on-site mechanobiological research.

## Introduction

Visualizing and quantifying cell structures like the cytoskeleton and nucleus are essential for understanding mechanobiology. Cells interrogate with its microenvironment via contractile cytoskeletal proteins, and this information is relayed to the nucleus via the mobilization of mechanosensitive factors [1,2]. For instance, cells on small adhesive islands exhibit reduced cytoskeletal contractility and decreased activity of mechanosensitive factors, such as YAP/TAZ (Yes-associated protein/transcriptional coactivator with PDZ binding motif) [3]. However, fluorescence staining, the standard approach to visualizing cellular structures, requires chemical dyes or fluorescence probes that are toxic to cells, allows limited simultaneous visualization of cellular structures, depends strongly on technical skills, and prevents long-term imaging of living cells [4].

Cell virtual staining uses artificial intelligence (AI) techniques to bypass the fluorescence staining steps and generates synthesized images for cellular structures of interest [5–9]. To date, cellular structures can be virtually stained from various microscopies [10–16]. While proof-of-concept virtual staining for specific cells exists, generalizing trained AI models to new, unseen cell types remains challenging [17–19]. Simply applying a trained model to new data often yields suboptimal performances [20]. Meanwhile, retraining models from scratch requires substantial labeled training data, which may not always be readily available. Therefore, there is an unmet need for a cell virtual staining toolbox with high generalization ability.

**Fig. 1.**
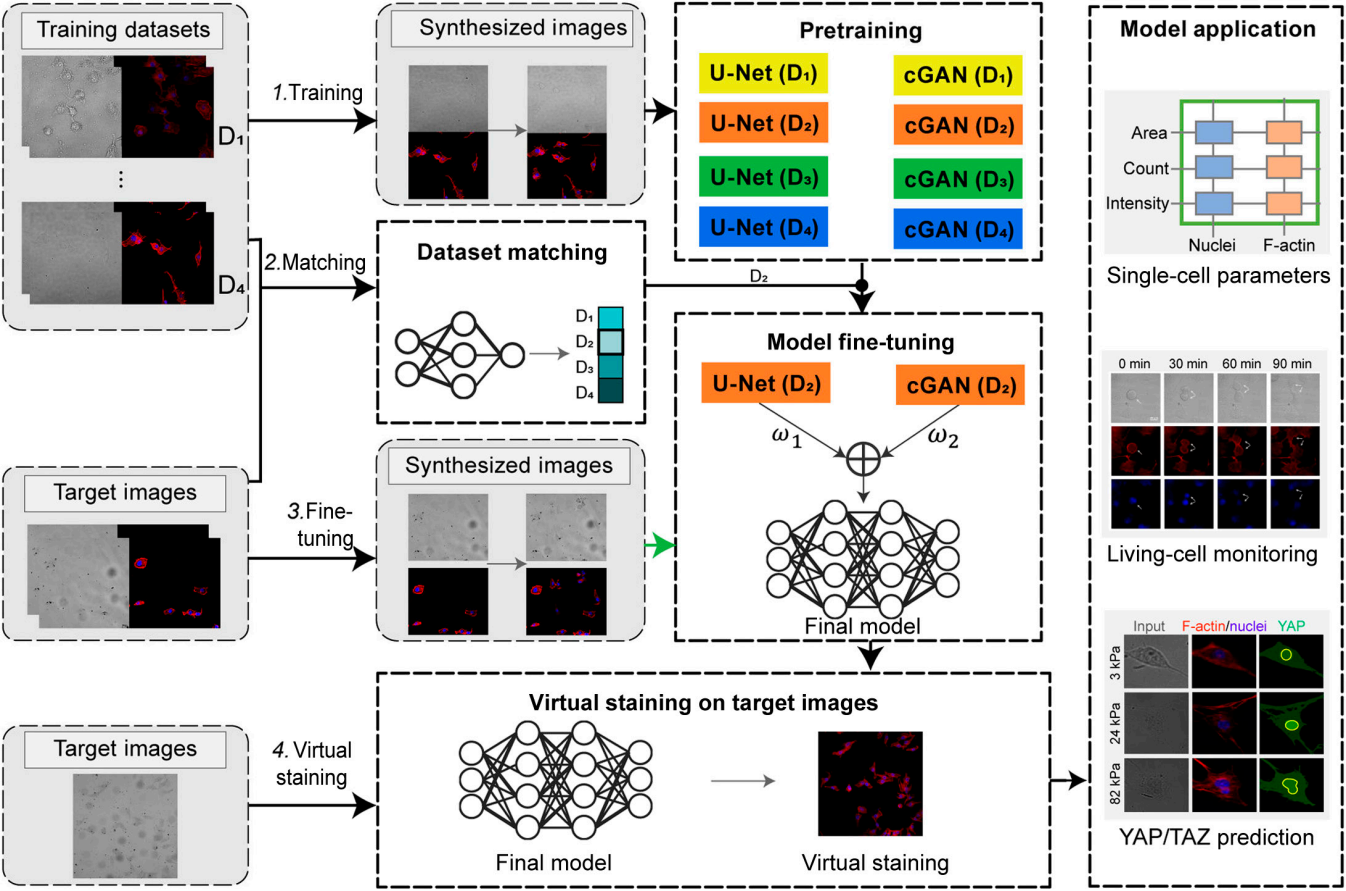
The framework of the generalizable cell virtual staining toolbox based on few-shot transfer learning. We created an ensemble of pretrained models with different data properties (step 1). The target images were automatically matched to one of the pretrained model (step 2). The matched pretrained model is fine-tuned using the target images (step 3). Virtual staining is performed for the target images, and the mechanobiological relevant single-cell parameters, cell mechanobiological status, and long-term monitoring of living cells are provided (step 4).

We develop a fully automatic cell virtual staining toolbox (termed CellVisioner) that can generalize to new data with minimal user input required (Fig. 1). We adopt few-shot transfer learning to drastically reduce the demand for user training data [21,22]. Specifically, instead of simply pretraining the model on one dataset and fine-tuning it on another, we improve the performance by allowing the model to start from a pretrained model with similar data properties. In the first phase, an ensemble of pretrained models is created from local datasets with diverse image properties. In the second phase, the target dataset is automatically matched to one of the pretrained models, allowing a fine-tuning procedure to start from a pretrained model with similar image properties. We demonstrate this toolbox on the virtual staining of F-actin and nuclei of various cells. We further demonstrate the toolbox’s ability to predict the mechanobiological status of cells and perform lone-term observation of living cells directly from the label-free input. Our strategy provides a generalizable framework for machine learning with limited domain-specific data. CellVisioner can be a powerful toolbox for on-site cellular mechanobiology analysis.

## Results

### Preparing training datasets with diverse image properties

The cell image datasets are highly diverse in terms of cell morphology and culture density. To address this issue, we created a number of local datasets with diverse cell morphology. Specifically, our local datasets included 2 types of cells with typically different cell morphologies, including human umbilical vein endothelial cells (HUVECs; i.e., round and textured shape) and NIH 3T3 (i.e., protrusive and irregular shape). For each type of cell, 2 widely used magnifications (i.e., 10× and 40×) were included, resulting in 4 datasets (i.e., HUVEC 40×, HUVEC 10×, NIH 3T3 40×, and NIH 3T3 10×). The training images consist of pairs of differential interference contrast (DIC) image and fluorescence labels for F-actin and nuclei. Details of the local datasets are provided in Section S1.

To further increase the diversity of training data, we created synthesized images of varying cell density and morphology. To do this, single cells were isolated from the original training images (i.e., DIC and corresponding fluorescence images) and slightly deformed to simulate the morphological changes of cells. Then, these isolated cells were randomly picked and seamlessly pasted on a background image with varying cell densities using a Poisson-blending data augmentation (PBDA) algorithm (see Materials and Methods) [23]. The image synthesis procedure was only applied on the training dataset to increase data diversity, while the testing dataset was untainted. As a result, a total of 3,225 single cells were isolated from the local training dataset, and 5,600 simulated images were generated.

### Creating an ensemble of pretrained models for cell virtual staining

**Fig. 2.**
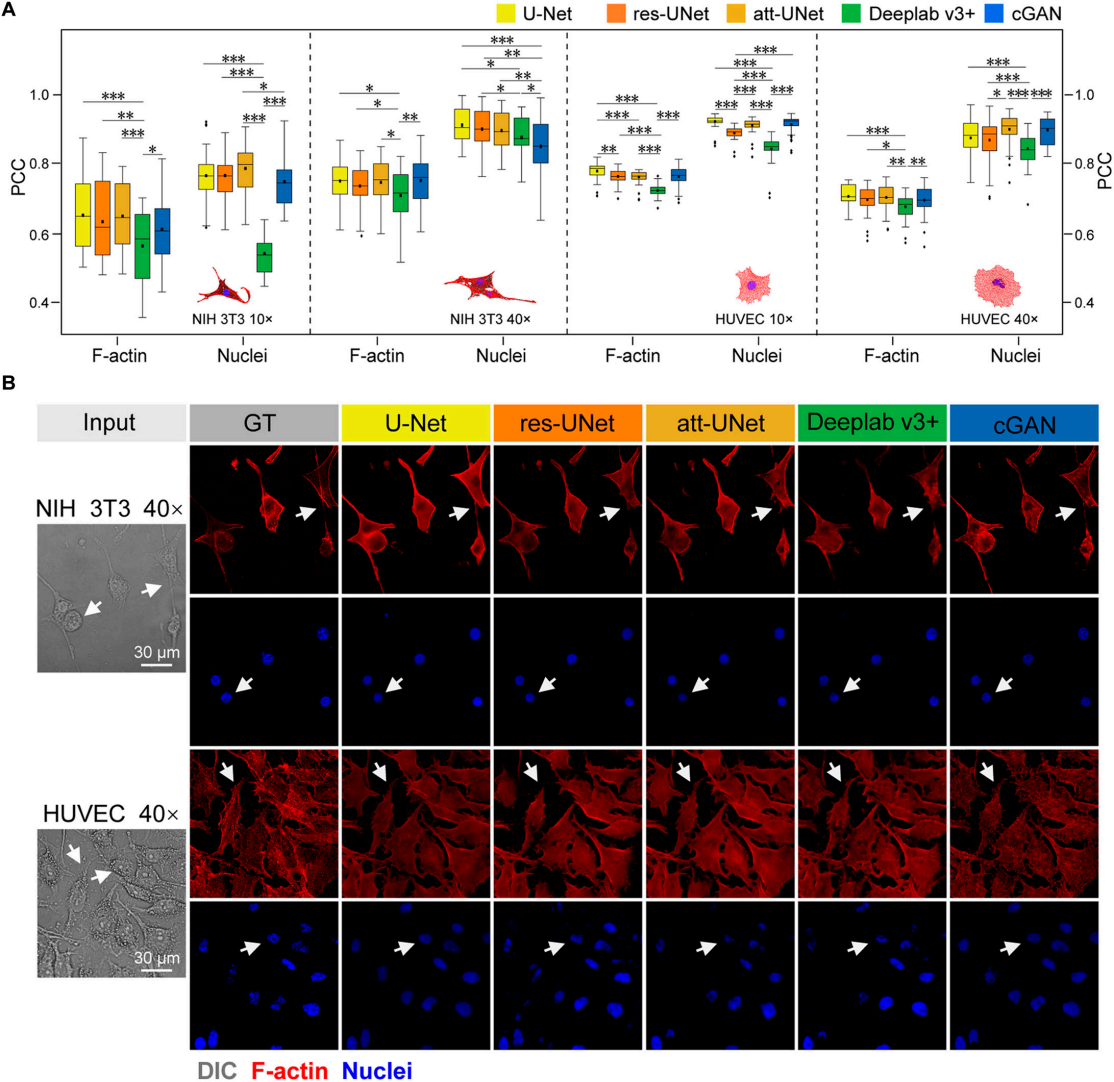
Performance of the pretrained models, including U-Net, res-UNet, att-UNet, DeepLab v3+, and cGAN. (A) PCC of the pretrained models on different datasets. The experiment was repeated 3 times on the same test set. Statistical significance levels obtained by 2-tailed unpaired Student’s *t* test: *P < 0.05; **P < 0.01; ***P < 0.001. (B) Visualized results of different models. U-Net and cGAN were able to provide finer details for F-actin.

A number of AI models have been adopted for cell virtual staining, the performances of which highly depend on the dataset properties [24]. Thus, we first compared the performances of several widely used AI models on our local datasets. Specifically, we evaluated 3 backbone models (i.e., vanilla U-Net, DeepLab v3+, and cGAN) and 2 popular add-on modules for U-Net (i.e., res-UNet and att-UNet) on our 4 established local datasets (i.e., HUVEC 40×, HUVEC 10×, NIH 3T3 40×, and NIH 3T3 10×). Vanilla U-Net and cGAN showed consistently higher performance in terms of Pearson correlation coefficient (PCC) on all datasets, while DeepLab v3+ showed consistently lower performance on all datasets (Fig. 2A). Attention and residual modules of U-Net did not show consistent improvements compared with vanilla U-Net. From visualized virtual staining results on HUVEC 40× and NIH 3T3 40× (Fig. 2B), we observed that U-Net and cGAN were able to provide finer details for F-actin. Thus, we chose vanilla U-Net and cGAN as the candidate models for further study. So far, we have created an ensemble of pretrained models (i.e., U-Net and cGAN) for the local datasets.

### Fine-tuning the pretrained models on unseen target dataset

The pretrained models need to be fine-tuned for the unseen target dataset. We collected human breast cancer cell line (i.e., MDA-MB-231) of different magnifications (i.e., 20× and 40×) as target datasets. To further increase the diversity of target datasets, we also recruited a set of induced pluripotent stem cell (iPSC) images from a public resource [10]. The number of training images for MDA-MB-231 20×, MDA-MB-231 40×, and iPSCs were 216, 336, and 32, respectively. More details regarding target datasets are provided in Section S2.

**Fig. 3.**
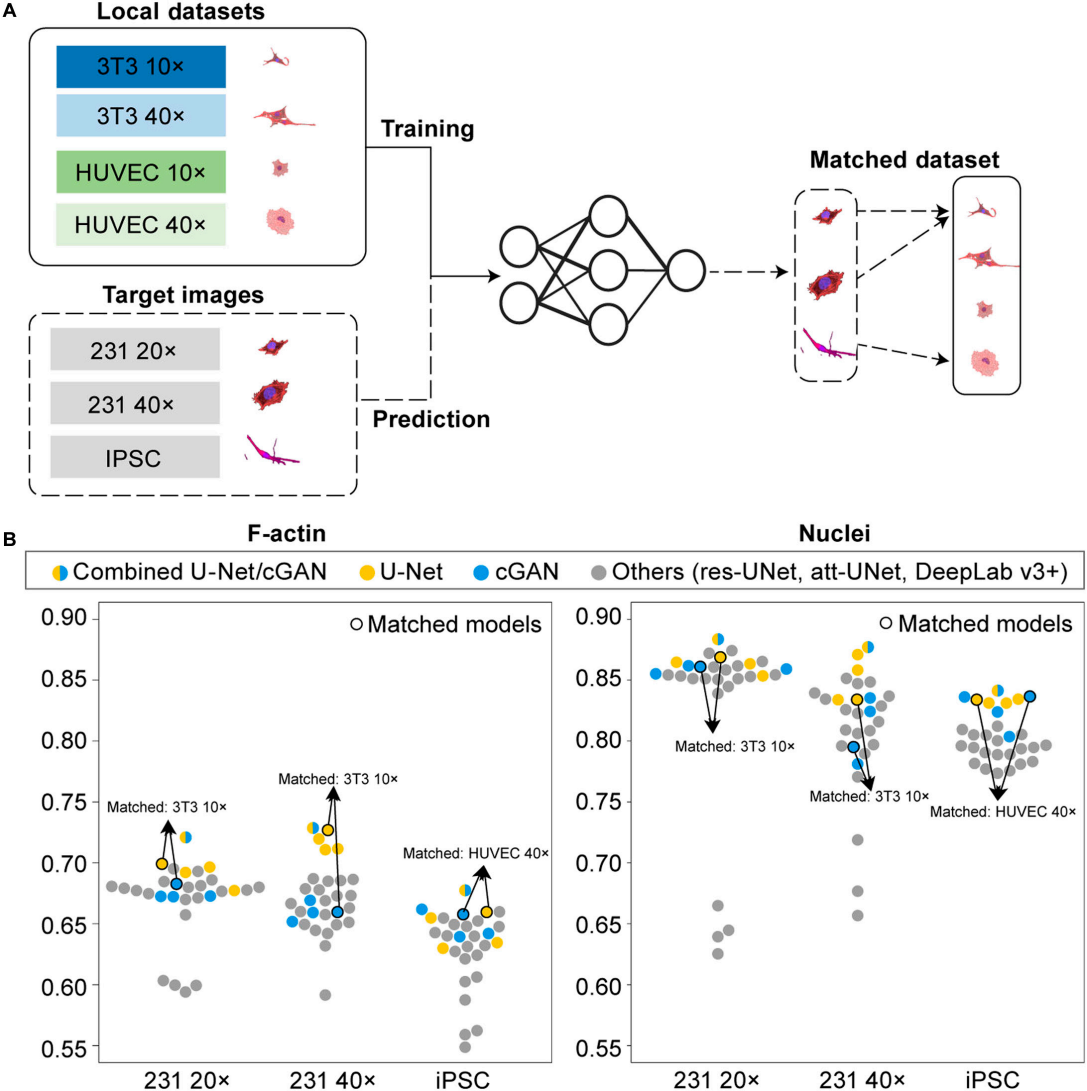
The unseen target image is matched to one of the local datasets, and the corresponding pretrained models are used for fine-tuning. (A) A supervised neural network is applied for dataset matching. (B) The performance of the fine-tuned U-Net, cGAN, the combined U-Net/cGAN model, and the other models on 3 types of target images (i.e., MDA-MB-231 20×, MDA-MB-231 40×, and iPSC). The yellow dots denote the U-Net models pretrained using the 4 local datasets. The blue dots denote the cGAN models pretrained using the 4 local datasets. The gray dots denote the other pretrained models (i.e., res-UNet, att-UNet, and DeepLab v3+). The black circle denotes the matched models.

The first step is to find the best matched models for the unseen target datasets. To do this, we trained a supervised VGG16 to match the target datasets to one of the local datasets (Fig. 3A) [25]. The matched datasets for MDA-MB-231 20×, MDA-MB-231 40×, and iPSCs were NIH 3T3 10×, NIH 3T3 10×, and HUVEC 40×, respectively. A fine-tuning step was followed starting from the pretrained models of the best matched dataset. In Fig. 3B, the yellow/blue dots with a black circle denote the matched models, while the yellow/blue dots without the black circle denote the unmatched models. First, compared with the unmatched models, the matched models generally provided a better performance. However, it is also observed that in some cases (i.e., MDA-MB-231 40×), the matched model showed a lower performance. Second, a further performance gain was achieved using a model combination strategy. The optimal combination ratios were (for both F-actin and nuclei) 0.5:0.5, 0.3:0.7, and 0.5:0.5 for MDA-MB-231 20×, MDA-MB-231 40×, and iPSCs, respectively (see Section S3). Thus, we chose the 0.5:0.5 combined U-Net/cGAN as the final model.

**Fig. 4.**
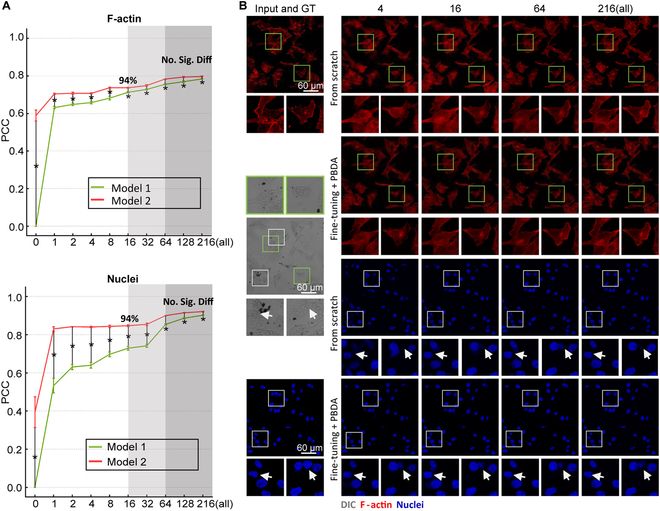
The influence of the number of training images. The target images are MDA-MB-231 20×. (A) Comparison of different training strategies. Model 1 was trained from scratch using the MDA-MB-231 20× dataset (green line). Model 2 was pretrained using the NIH 3T3 10× dataset and fine-tuned using the MDA-MB-231 20× dataset (red line). The experiment was repeated 5 times on the same test set. Statistical significance levels obtained by 2-tailed unpaired Student’s *t* test: *P < 0.05. (B) Visualized results of virtual staining with different training strategies.

Another key question here is how many images are required for fine-tuning from the user. In this respect, we studied the relationship between model performance and the number of fine-tuning images using MDA-MB-231 cells. The combined U-Net/cGAN model was trained using 2 different strategies. Model 1 was trained from scratch using the MDA-MB-231 20× dataset, and the best performance was achieved with all 216 training images (Fig. 4A, green line). Model 2 was pretrained using the NIH 3T3 10× dataset, which consists of 284 pairs of DIC and fluorescence images. Then, it was fine-tuned using the MDA-MB-231 20× dataset (Fig. 4A, red line). Model 2 demonstrated a performance of no significant difference with the best performance of model 1 when using 64 images and achieved 94% of the best performance with only 16 images. Visual results also indicated that model 2 was able to present more fine details (Fig. 4B).

### Extracting quantitative single-cell parameters

**Fig. 5.**
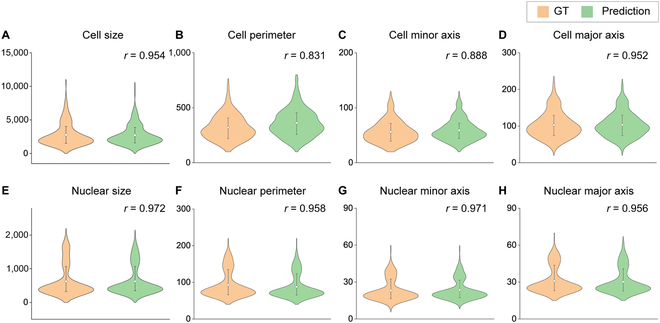
Single-cell parameters are extracted from the virtually stained image and compared with the ground truths using PCC (*r*). (A to D) Comparisons of the cellular (F-actin) parameters extracted from the ground truth and the predictions, including (A) cell size, (B) cell perimeter, (C) cell minor axis, and (D) cell major axis. (E to H) Comparison of the nuclear parameters extracted from the ground truth and the predictions, including (E) nuclear size, (F) nuclear perimeter, (G) nuclear minor axis, and (H) nuclear major axis.

We extracted several mechanobiological-relevant single-cell profile parameters from the virtually stained image and compared them with the ground truth. The model was pretrained using the local NIH 3T3 10× dataset and was fine-tuned using 16 MDA-MB-231 images for F-actin and nuclei, respectively. A set of 213 single cells were isolated from the virtual staining images. We calculated 8 mechanobiological-relevant cellular and nuclear parameters (i.e., mean area, perimeter, minor axis, and major axis of cell and nucleus) (Fig. 5). Most of the predicted structural parameters agreed well with their ground truth (PCC *r* larger than 0.8), showing that our virtual staining toolbox is capable of providing accurate structural quantification at single-cell level.

### Predicting mechanobiological state at the single-cell level

**Fig. 6.**
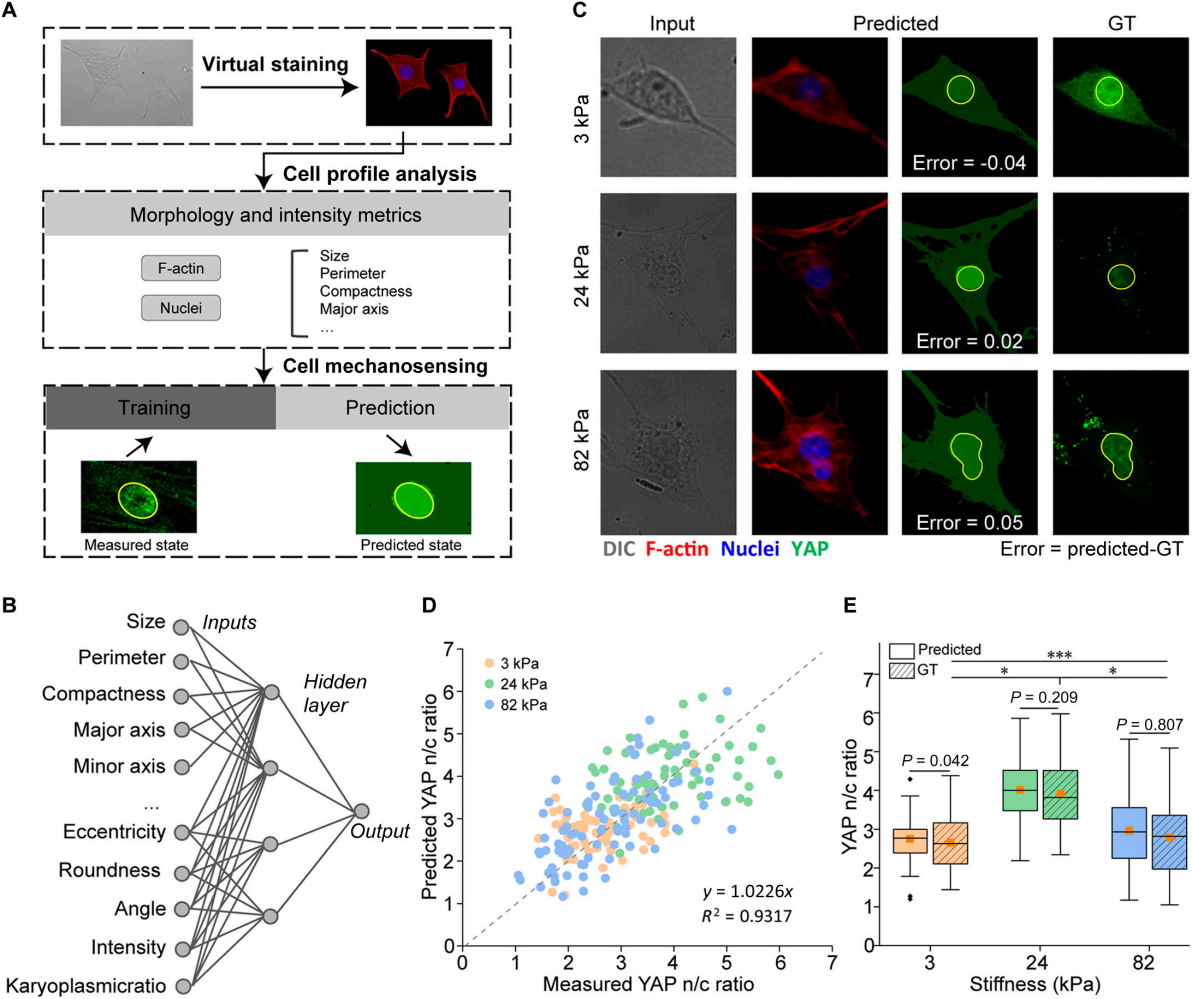
The single-cell YAP n/c ratio is predicted directly from the label-free cell images. (A) Workflow of the prediction procedure. The virtually stained F-actin and nuclei are obtained from DIC images, followed by the extraction of the cellular and nuclear parameters at a single-cell level. A neural network is then trained to predict YAP n/c ratio. (B) The neural network consisted of 17 inputs and 3 hidden layers (256, 256, 4) to predict the n/c ratio of YAP. (C) Visualized results of the DIC image, predictions, and ground truth. (D) Scatter plot of predicted and measured YAP n/c ratio under 3 different stiffnesses. (E) Box plot of the YAP n/c ratio under different stiffnesses. Statistical significance levels obtained by 2-tailed unpaired Student’s *t* test: *P < 0.05; **P < 0.01; ***P < 0.001.

The mechanobiological state of a cell is predictable from its cellular and nuclear morphology [26]. Here, we explored the feasibility of predicting the YAP activation through its nuclear/cytoplasmic ratio (n/c ratio) from the virtually stained F-actin and nuclei of human breast carcinoma cell line (MCF-7). Cells were cultured on gels with 3 different stiffness (i.e., 3, 24, and 82 kPa). The label-free DIC and corresponding YAP staining images were captured, resulting in a total of 232 image pairs (72 for 3 kPa, 80 for 24 kPa, and 80 for 82 kPa, respectively). The model was pretrained using the local HUVEC 40× dataset and was fine-tuned using 16 MCF-7 images for F-actin and nuclei, respectively (Fig. 6A). Virtual staining of nuclei and F-actin was generated from the label-free cell images, and the cellular and nuclear parameters were calculated. We extracted the 8 basic parameters as described in previous section and further included a set of advanced parameters, including compactness, eccentricity, angle of major axis, mean intensity of cell and nucleus, and the nucleocytoplasmic ratio. A supervised 3-layer neural network (256, 256, 4) was trained to predict the YAP n/c ratio using the extracted single-cell parameters (Fig. 6B). Our predicted staining matched well with the true chemical staining (Fig. 6C). The good consistency of the overall predicted YAP n/c ratio and the ground truth was confirmed further by *R*^2^ error assessment (*R*^2^ = 0.9317) (Fig. 6D). The comparison of predicted and true value of YAP n/c ratio in each stiffness group showed good consistence as well (Fig. 6E). Interestingly, our prediction implies the existence of optimal stiffness (24 kPa) for YAP activation, which is also observed in relevant experimental studies [27].

### Monitoring living cells using the virtual staining toolbox

**Fig. 7.**
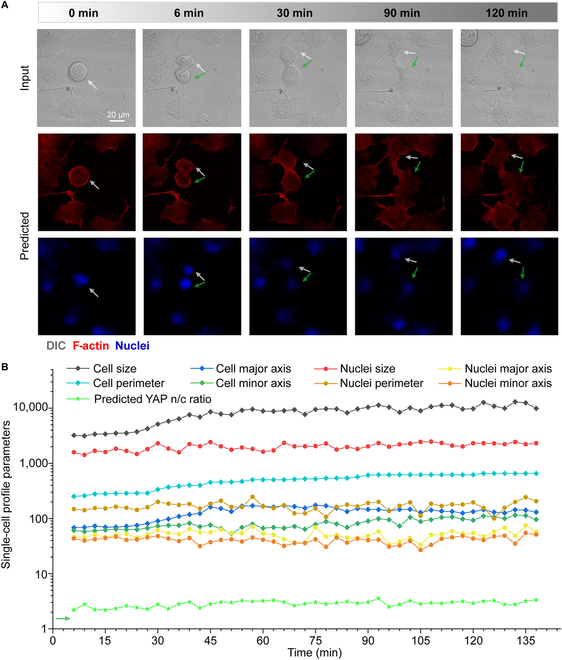
Monitoring of label-free living cells is conducted. To illustrate the value of virtual staining on the monitoring of living cells, we cultured MCF-7 cells and acquired multiple label-free DIC images in a fixed field. (A) The virtually stained F-actin and nucleus images were predicted from the label-free DIC images. In continuous observations, living cell life phenomena, such as cell division and migration, are captured. (B) The single-cell parameters of the living cells were continuously measured.

Our virtual staining toolbox allows for continuous monitoring of F-actin and nuclei with label-free input. To demonstrate this, we cultured MCF-7 cells and made continuous observations over time. Virtual staining of F-actin and nuclei was obtained from the label-free DIC images (Movie S1). Cells underwent mitosis and were constantly migrating during our observation. As shown in Fig. 7A, the pseudopod grew out and pulled the cell body to move during migration process. It was evident that the nuclei got rounder and then slowly separated from one to 2 (white and green arrows). Mechanobiological relevant cellular parameters and YAP n/c ratio were continuously observed in the video (Fig. 7B). During the first half hour of division, the area, perimeter, and major axis of cell increased substantially, whereas the morphology of the nucleus did not show obvious changes.

## Discussion

In this study, we developed CellVisioner, an AI-based cell virtual staining toolbox that can achieve highly generalized performance with minimal user training data. A major challenge in developing AI models for biomedical research is the limited availability of training data, especially for new experimental conditions or cell types [28]. Our 2-phase transfer learning approach overcomes this limitation by pretraining models on diverse local datasets before fine-tuning on small user datasets. We chose 2 cell lines with disparate cell morphology, used 2 magnifications, and created synthesized images to simulate cells with varying image properties. Furthermore, fine-tuning is implemented to customize the pretrained model specifically for the target cell images. This significantly enhances the performance on new target data with reduced demand for user-provided examples. With our 2-phase transfer learning strategy, the required training data from user have been largely reduced. CellVisioner can achieve an acceptable performance (94% of the best performance) with only 16 target images of a size of 512 × 512 pixels. A microscopy typically has an output image size of 1,024 × 1,024 pixels or larger, which means only 4 target images are needed to fine-tune the model for user-specific application. To further evaluate its generalization ability, CellVisioner was tested on 2 more cell lines (i.e., A549 and WM115) (Section S4).

Mechanobiology focuses on how cell senses and responses mechanical stimuli, which is reflected in cell activities like growth, migration, and differentiation. Breast cancer MDA-MB-231 cell was chosen because it is one of widely used cell lines to study migration and metastasis of cancer cell in circumstances of mechanical stimulation [29,30]. The iPSCs, which are derived from reprogrammed adult cells, can sense mechanical stimuli and consequentially differentiate into different cell lineages, such as cardiomyocytes and neurons [31]. For example, iPSC can model cardiac contractile mechanical output more robustly when cell shape is manipulated into physiological shapes via cell micropatterning [32]. Virtual staining and quantifying the mechanical output of these cells can greatly facilitate related mechanobiology researches. We have shown that our virtual staining toolbox can capture the complex morphological features of the cytoskeleton and nucleus of these cells. We have further shown that, by extracting a multitude of cellular and nuclear parameters from the virtually stained images, CellVisioner can predict the YAP n/c ratio directly from the label-free cell images. The translocation of YAP reflects the degree of cell response to mechanotransduction (e.g., extracellular matrix stiffness, shear stress, viscoelasticity, and plasticity) [33–35].The capacity to predict mechanobiological status directly from living cell images could make CellVisioner a useful high-content screening tool for studying mechanosignaling pathways or drug discovery.

We also demonstrated that, taking the label-free cell images as input, our toolbox allows for a prolonged observation for the cellular and nuclear activities of living cells. Many cellular studies require prolonged observation of the cell activities, such as growth, division, and migration. The ability to provide virtual stains for living cells presents many opportunities. For example, CellVisioner could be used to track the speed and direction of cell migrations, thus facilitating the study of the mechanism of mechanical factors (e.g., extracellular matrix stiffness [36,37] and shear stress [38,39]) during physiological and pathological processes. These applications have shown the toolbox’s capability to significantly improve the throughput for mechanobiological analysis.

To increase the accessibility of CellVisioner, we provided a web platform where users can easily create virtual staining models for their own needs. The online platform is available at http://bioimganalysisai.org.cn/ (Movie S2). We have also released the pretrained models and local datasets for researchers in the community(https://github.com/xjtu-mia/CellVisioner).

The toolbox still has some limitations. The current single-cell parameter extraction and YAP/TAZ prediction require manual segmentation of single-cell images. Automatic cell segmentation may further improve the analysis efficiency. Although the demand for user training data has been largely reduced, the current strategy still requires fluorescence label images from users for fine-tuning, limiting the toolbox’s ability for quick on-site usage. The advance of other techniques, such as domain adaptation, presents an opportunity for zero-shot learning and may overcome this limitation in the future.

An important future direction is expanding the structural targets that can be virtually stained by CellVisioner. While we currently demonstrate the virtual staining of F-actin and nuclei, other structures like intermediate filaments, endoplasmic reticulum, or mitochondria could be incorporated using appropriate training data. A larger diversity of targets would make CellVisioner more applicable to a wider range of mechanobiological questions.

In summary, we have developed a cell virtual staining toolbox with high generalization ability based on few-shot transfer learning, which avoid the need for heavy training from users. The proposed toolbox was able to provide accurate cellular structure quantification at single-cell level. In addition, we have also shown its capability to predict the mechanobiological status of a cell and perform long-term observation of living cells directly from label-free inputs. CellVisioner would be a powerful tool to facilitate on-site mechanobiological research.

## Materials and Methods

### Image acquisition

#### Cell preparation and chemical staining

NIH 3T3, HUVECs, A549, and WM115 were plated separately at 75-cm^2^ flasks (Thermo Fisher Scientific, USA) in Dulbecco’s modified Eagle medium:nutrient mixture F-12 (DMEM/F-12; Hyclone, USA), containing 10% fetal bovine serum (FBS; Gibco, USA), and 1 % penicillin-streptomycin (Pen-Strep; Gibco, USA) at 37 °C and 5% CO_2_. MDA-MB-231 cells were cultured in Leibovitz’s L-15 medium containing sodium bicarbonate (1.18 g/l), l-glutamine (1.3 g/l), and streptomycin (0.08 mg/ml) at 37 °C and 5% CO_2_.

Cells were seeded at a density of 1 × 10^5^ cells/ml in 3.5-cm^2^ petri dish (Thermo Fisher Scientific, USA) and were cultured in DMEM/F-12 medium containing 10% FBS and 1% Pen-Strep overnight. Samples were fixed in 4 % paraformaldehyde for 20 min and washed 3 times in phosphate-buffered saline (PBS) for 5 min before penetrating samples with 0.5% Triton X-100 (Sigma-Aldrich, USA) for 15 min and washed 3 times in PBS for 5 min. To visualize F-actin and nuclei, the samples were stained by Alexa Fluor 488 488-labeled phalloidin for 1 h and 4′,6-diamidino-2-phenylindole (1 μg/ml; D9542, Sigma-Aldrich, USA) for 10 min.

#### Cell culture and YAP/TAZ staining

The proportion of acrylamide and *N*,*N*′-methylenebisacrylamide in polyacrylamide hydrogel was adjusted to make hydrogel with stiffnesses of 3, 24, and 82 kPa. Polyacrylamide hydrogel was incubated with rat tail collagen at 4 °C for 12 h before use. MCF-7 cells were inoculated on collagen treated hydrogels for 24 h. For YAP staining, the cells were fixed with 4% paraformaldehyde at room temperature for 15 min, transfused with 0.5% Triton X-100 PBS, sealed with 2% bovine serum albumin PBS, and incubated with primary antibody. The antibody used for immunofluorescence was YAP (1:500; ab205270, Abcam, UK). After washing, the cells were incubated with the second antibody.

#### Living cell observation

MCF-7 cells were inoculated at 20-mm nunc glass bottom dish (NEST, China) in DMEM/high glucose (Corning, USA), containing 10% FBS (Gibco, USA) and 1% Pen-Strep (Gibco, USA) at 37 °C and 5% CO_2_. After 5 h of cultivation, label-free samples were placed in a live-cell workstation for tracking. Several coordinates were set as specific automatic scanning points. To avoid the loss of focus caused by cell growth or sample floating, we set the number of z-stack in the range of 8 to 10. The lens automatically scanned for more than 3 h, with lens scanning every 3 min.

#### Image data acquisition

Image data were taken by an Olympus FV3000 laser scanning confocal microscope. Excitation and emission wavelengths were as follows for fluorescence channels: nuclei (excitation: 405 nm; emission: 461 nm) and F-actin (excitation, 561 nm; emission, 586 nm). The cells were imaged with a DIC z-stack of 5 images with 2.5-μm intervals. And image data are available in 3 magnifications: 10×/0.4 DIC objective, 20×/0.75 DIC objective, and 40×/0.6 DIC objective. The dimensions of the above images are 1.243, 0.621, and 0.311 μm/pixel, and the resolution is 1,024 × 1,024 pixels.

### Generation of synthesized training images

Synthesized training images are generated on the basis of an instance-level data augmentation algorithm [23]. Single cells are identified in the binarized fluorescence image of F-actin as connected components, which are then used as masks to extract single cells from the label-free image and nuclear label image. The label-free image and corresponding fluorescence images of single cells are stored in pairs in a source pool. A background image is randomly picked from the original training set. Then single-cell images (a maximum number of 20) are randomly selected from the source pool and seamlessly pasted on the background image using the PBDA algorithm [40]. A rescaling of size between 0.8 and 1.2 is applied before pasting to simulate the deformation of cells. For each cell image, the pasting location is randomly selected. If overlapping happens, the pasting will stop and move to the next random location with a maximum of 100 attempts. False positives are reported to be introduced by Poisson blending in the literature study [23], in which Poisson blending is used for the synthesis of pathological lesions with diverse size and shape. On the other hand, the instance in our study is cell, which has a more regular shape and size. Thus, artifacts introduced by Poisson blending were not observed in the current study. Additional details of the image synthesis algorithm are provided in Section S5.

### AI models and pretraining

We implement 5 popular AI models to learn the transformation from the label-free cell image to the fluorescence labels, including vanilla U-Net [41], 2 derivative models of U-Net (i.e., att-UNet and res-UNet) [42,43], DeepLab v3+ [44], and cGAN. More details regarding the design of AI models are provided in Section S6.

All models were implemented using Python version 3.8.0, with TensorFlow framework version 2.4.0. Model training and testing were performed using an NVIDIA RTX3090 GPU. Hyperparameters, including optimizer, batch size, loss function, learning rate, and decay strategy, are optimized with regard to each model (see Section S7). The models take a *z*-axis stack of 512 × 512 pixels as input images. The output is a single-channel fluorescence image with 512 × 512 pixels. All models are trained and evaluated for nuclei and F-actin separately.

### Fine-tuning and model combination

Directly applying a trained model to unseen data may result in suboptimal performances; thus, we propose a few-shot transfer learning to improve the generalization ability of the model. In the first phase, we establish an ensemble of pretrained models using our local datasets. In the second phase, the unseen target images are automatically matched to one of the pretrained model for fine-tuning. We developed a supervised VGG16 to match the unseen target images to one of the local datasets [25].

The selected pretrained models are then fine-tuned for the target dataset. For U-Net, we fine-tune all layers of the pretrained model with a reduced learning rate to avoid drastic changes in the model. For cGAN, we fine-tune the weights of all layers except the last layer of the discriminator with a reduced learning rate [45]. The last layer is reinitialized for the target dataset. More details of the fine-tuning procedures are provided in Section S7.

### Evaluation metrics

We use the patch-wise PCC to evaluate the performance of the models. Specifically, PCC is used to quantify the prediction quality of nuclei and F-actin. It computes the statistical correlation between the predicted label image and the chemical label image patches and can quantify the pixel-level similarity on the fine cellular features. The PCC between the prediction *X* and ground truth *Y* is computed as:

$$\mathrm{PCC}=\frac{\sum_{i=1}^{N}\left(X_{i}-\bar{X}\right)\left(Y_{i}-\bar{Y}\right)}{\sqrt{\sum_{i=1}^{N}{\left(X_{i}-\bar{X}\right)}^{2}}\sqrt{\sum_{i=1}^{N}{\left(Y_{i}-\bar{Y}\right)}^{2}}+\varepsilon }$$

where *ε* is a small regularizer to prevent zero denominator. The value of PCC ranges from −1 to +1, indicating from the total negative correlation (−1) to the total positive correlation (+1).

## Data Availability

The local datasets are available for researchers in the community (https://github.com/xjtu-mia/CellVisioner).

## Supplementary Materials

Supplementary 1 Figs. S1 to S6 Tables S1 to S6 Sections S1 to S7
