# Supplementary material for: CellVisioner: A Generalizable Cell Virtual Staining Toolbox based on Few-Shot Transfer Learning for Mechanobiological Analysis
Source: Research (Wash D C). 2023 Dec 5;6:0285. doi: 10.34133/research.0285 (PMC10907024; doi:10.34133/research.0285)
Supplement: Supplementary 1 — Figs. S1 to S6 Tables S1 to S6 Sections S1 to S7 [file research.0285.f1.zip › APowerfulVirtualStainingToolbox-SI-Research-Markedup.docx]

Supplementary Materials for

**CellVisioner: A Generalizable Cell Virtual Staining Toolbox based on Few-Shot Transfer Learning** **for Mechanobiological Analysis**

Xiayu Xu^a,b^, Zhanfeng Xiao^a,b^, Fan Zhang^a,b^, Changxiang Wang^a,b^, Bo Wei^a,b^, Yaohui Wang^a,b^, Bo Cheng^a,b^, Yuanbo Jia^a,b^, Yuan Li^a,b^, Bin Li^a,b^, Hui Guo^c^, Feng Xu^a,b^*^*^*

*^a^ The Key Laboratory of Biomedical Information Engineering of Ministry of Education, Xi’an Jiaotong University, Xi'an 710049, P.R. China*

*^b^ Bioinspired Engineering and Biomechanics Center (BEBC), Xi’an Jiaotong University, Xi'an 710049, P.R. China*

*^c^ Department of Medical Oncology, The First Affiliated Hospital of Xi’an Jiaotong University, Xi’an 710061, P.R. China*

*^*^ Corresponding author:* fengxu@mail.xjtu.edu.cn

**Section S1. Details of the local datasets**

After NIH 3T3 and human umbilical vein endothelial cells (HUVECs) were cultured and stained, cell image data were taken by an Olympus FV3000 Laser scanning confocal microscope. Excitation and emission wavelengths are as follows for fluorescence channels: nuclei (ex: 405nm, em: 461nm), actin (ex: 561nm, em: 586nm). The cells were imaged with a DIC z-stack of five images with 2.5 microns intervals. And image data are available in two magnifications: 10×/0.4 DIC objective and 40×/0.6 DIC objective. The resolution of the above images is 1.243 μm/pixel and 0.311 μm/pixel, respectively. The original image size is 1024×1024 pixels, which is split into four images of size 512×512 pixels as model input. The training, validation, and test sets are divided approximately with a proportion of 7:1.5:1.5 (**Table S1**). For the label-free DIC images, contrast limited adaptive histogram equalization is performed as preprocessing. Maximum intensity projection along the z-axis and median filtering is performed for the fluorescence images.

**Section S2. Details of target datasets**

We collected human breast cancer cell line (*i.e.*, MDA-MB-231) of two different magnifications (*i.e.*, 20× and 40×) and induced pluripotent stem cells (iPSC) as target datasets. The original images have a size of 1,024×1,024 pixels and are split into four images of size 512×512 pixels. The more details are shown in **Table S2**.

**Section S3. Performances of the combined U-Net/cGAN model**

A weighted combination of U-Net and cGAN from 0.1:0.9 to 0.9:0.1 with an incremental step of 0.2 was explored. The combination results of U-Net and cGAN are showed in **Table S3 and Table S4**.

**Section S4. Generalizing CellVisioner to different cell lines**

To further evaluate the generalization ability, the proposed approach was generalized to two more cell lines (*i.e.*, A549 lung carcinoma cells and WM115 melanoma cells). The combined U-Net/cGAN model was pretrained with HUVEC 40× and 3T3 40×, respectively, and was fine-tuned using 16 target images of size of 512×512 pixels. The performance was evaluated on a separate testing dataset of 7 images in terms of PCC. The results are shown in **Fig. S1**. For A549, the averaged PCC is 0.691 and 0.863 for F-actin and nuclei, respectively. For WM115, the averaged PCC is 0.680 and 0.774 for F-actin and nuclei, respectively.

**Section S5. Description of the cell-level data augmentation algorithm**

In the experiment, the training images are generated based on a cell-level data augmentation algorithm known as Poisson-blending. The flowchart is shown in **Fig. S2**.

**1.** Cell source pool construction

First, on the training set, we overlay z-stack of 5 actin fluorescent images as anchor to identify cell positions and contours, which guide the cropping of cell patches in DIC, actin, and DNA fluorescent images from training sets. The cell patches library L is constructed as:

$$\begin{aligned} \boldsymbol{L}=\left\{ a_{0},a_{1}\ldots a_{n1},b_{0},b_{1}\ldots b_{n2},c_{0},c_{1}\ldots c_{n3} \right\}\#\left( 1 \right) \end{aligned}$$

where a, b, c indicated a pair of images for DIC, F-actin, and nuclei, respectively. A total of 3,225 single cells were isolated from the local training datasets and 5,600 simulated images were generated.

A background image is randomly picked from the original training set. Then a random number (with a maximum of 20) of single-cell images are randomly selected from the source pool and seamlessly pasted on the background image using the Poisson-blending algorithm. For each cell image, the pasting location is randomly selected and, if overlapping happens, the pasting will stop and move to the next random location with a maximum of 100 attempts.

**2.** Image pasting by Poisson blending algorithm

We used a Poisson blending algorithm to seamlessly paste the cell patches into background images. The core mathematical principle of this method is a Poisson partial differential equation with Dirichlet boundary conditions, which specifies the Laplace operator of the unknown function in the region of interest, and solves the pixel gray value of the fusion region by constructing a coefficient matrix. Suppose that there is a closed subregion Ω on the two-dimensional image plane S, whose boundary is ∂Ω, V is the gradient vector field defined on Ω, $f$ and $f^{*}$ are a unknow and known scalar function defined on Ω, V is the guidance vector field. then $f$ takes the interpolation function guided by V in Ω, that is, to solve the extreme value problem:

$$\begin{aligned} \min_{f}\iint_{\Omega} \left| \nabla f-V \right|^{2},\left. f \right|_{\partial\Omega}=\left. f^{*} \right|_{\partial\Omega}\#\left( 2 \right) \end{aligned}$$

whose solution is the unique solution to the Poisson equation with Dirichlet boundary conditions:

$$\begin{aligned} \Delta f=\text{div}V, \left. f \right|_{\partial\Omega}=\left. f^{*} \right|_{\partial\Omega}\#\left( 3 \right) \end{aligned}$$

where Δ is Laplace operator, div is divergence operator. For digital images, the equation (2) can be expressed as:

$$\begin{aligned} \min_{f\left| \Omega\right.}\sum_{\left\langle\text{p,q} \right\rangle\cap\Omega\neq\emptyset} \left( f_{p}-f_{q}-V_{\text{pq}} \right)^{2}\#\left( 4 \right) \end{aligned}$$

where $\left\langle p,q \right\rangle$ is a pair of 4 connected adjacent pixels, $f_{p}$ and$f_{q}$ are the values of $f$ on p and q respectively, v $V_{pq}$is the projection of $V\left( \frac{p+q}{2} \right)$ on the oriented edge $\left\langle p,q \right\rangle$, and the boundary condition changes to$\forall_{p}\in\partial\Omega，f_{p}={f_{q}}^{*}$.

To generate uniformly distributed synthetic cell DIC and label datasets, cell patches and background cell images were randomly selected from the training set X and cell patches library L. The cell patches library was randomly rotated and flipped and rescaled before Poisson blending process. Therefore, the process can be described as:

$$\begin{aligned} O^{'}=PB\left( O,l_{i},\left( x_{c},y_{c} \right) \right), O\in X,l_{i}\in L,\left( x_{c},y_{c} \right)\in O\cap\bar{\varphi}\#\left( 5 \right) \end{aligned}$$

where $O^{'}$ and $O$ indicates synthetic images and background images. $l_{i}$ specifies DIC and its fluorescence images. $\left( x_{c},y_{c} \right)$ is the target center coordinates，$\bar{\varphi}$ is the location constraints imposed.

**Section S6. Details of the design of AI models**

1. **U-Net**

U-Net architecture consists of a contracting path to capture context and a symmetric expanding path to enables precise localization (**Fig. S3**) [1]. The contracting path consists of repeated application of two 3×3 convolutions, each followed by a rectified linear unit (ReLU) and a 2×2 max pooling operation with stride 2. The number of feature channels is doubled in our application. Each expansive step includes an up-sampling layer that doubles the size of feature maps using bilinear interpolation, and a convolutional block that reduces the number of feature channels. Skip connections between contraction and expansion paths are added. In the final layer, a 1×1 convolution is used to compress the number of feature channels to 1, thus enabling the transformation of information from the feature dimension to the image dimension. Dropout layers with dropout rate between 0.1 and 0.3 is used between two convolutional blocks to mitigate overfitting.

1. **Res-UNet**

The Res-UNet consists of an encoder-decoder architecture as the original U-Net. Residual blocks have been used to address the issue of gradient vanishing in deep convolutional networks **(Fig. S4)** [2]. The feature map is composed of a residual map and an identity map and the output is$y = F (x) + x$. Every residual unit contains two 3×3 convolutional layers and a skip connection to learn the residual features. Skip connections between contraction and expansion paths are added.

1. **Att-UNet**

For att-UNet, the attention gate (AG) is incorporated into the standard U-net network to enhance model accuracy and sensitivity to foreground pixels (**Fig. S5**) [3]. In the expanding path, four attention gates are used to extend the standard U-Net model. Additive attention gates are implemented through the skip connections before sequential processing of concatenation operation. During the process of backward propagation, the gradients originating from background regions can be reduced and the parameters depending on spatial regions relevant to a given task at each multi-scale level can be enhanced.

1. **DeepLab v3+**

DeepLab v3+ is a model originally proposed for semantic image segmentation [4]. To handle the problem of segmenting objects at multiple scales, this model employs atrous convolution to capture multi-scale context. We enrich contextual information using DeepLab v3+ to restore the boundaries of cellular structures.

1. **Conditional GAN (cGAN)**

The generator network of our cGAN model is the same as a U-Net model (**Fig. S6**) [5]. The discriminator attempts to distinguish the generated images from fluorescently-stained samples, classifying them as either real or synthetic data. The conditional aspect of the **c**GAN refers to the fact that the discriminator receives both the bright-field stack and the stained images as inputs. The discriminator in this work is constructed based on the patchGAN architecture, which consists of 4×4 convolutional, instance normalization, and LeakyReLU modules. The downsampling is performed by convolutional operations with a stride of 2, over a sequence of 5 such operations. By evaluating each local region of the generated image and averaging the discriminator output across all regions, the generator can better control the local details and produce sharper fluorescent details.

**Section S7. Implementation details of the training and fine-tuning**

We trained from scratch and fine-tuning on pretrained 3T3 10× U-Net and cGAN using increasing numbers of 231 20× images. The training, validation, and testing datasets contain 216, 56, and 56 images, respectively. The numbers of the training sets are 0, 1, 2, 4, 8, 16, 32, 64, 128, 216 (all images) images. Each experiment was repeated five times. The significant difference was calculated by GraphPad Prism-7 for unpaired students T-test. Implementation details of the model pretraining and fine-tuning are listed in **Table S5 and Table S6.**

**References**

1. Ronneberger, O., P. Fischer, and T. Brox. *U-Net: convolutional networks for biomedical image segmentation*. in *Medical Image Computing and Computer-Assisted Intervention - MICCAI 2015*. 2015.

2. He, K., X. Zhang, S. Ren*, et al.* *Deep residual learning for image recognition*. in *2016 IEEE Conference on Computer Vision and Pattern Recognition (CVPR)*. 2016.

3. Schlemper, J., O. Oktay, M. Schaap*, et al.*, Attention gated networks: Learning to leverage salient regions in medical images*.* *Medical Image Analysis*, 2019. **53** p. 197-207.

4. Chen, L.-C., Y. Zhu, G. Papandreou*, et al.* *Encoder-decoder with atrous separable convolution for semantic image segmentation*. in *Computer Vision - ECCV 2018*. 2018. Cham: Springer International Publishing.

5. Wang, T.C., M.Y. Liu, J.Y. Zhu*, et al.* *High-resolution image synthesis and semantic manipulation with conditional GANs*. in *2018 IEEE/CVF Conference on Computer Vision and Pattern Recognition*. 2018.

**Table S1. The details of training datasets.** The dataset consists of label free DIC and fluorescence images of NIH 3T3 and HUVEC cells, acquired at the magnifications of 10× and 40×.

| Cell type | Magnification | Resolution(μm/pixel) | Image Size | Image # (train/val/test) |
| --- | --- | --- | --- | --- |
| NIH 3T3 | 10× | 1.243 | 512×512 | 188/44/52 |
|  | 40× | 0.311 | 512×512 | 336/72/72 |
| HUVEC | 10× | 1.243 | 512×512 | 276/68/64 |
|  | 40× | 0.311 | 512×512 | 292/52/56 |

**Table S2. The details of target datasets.**

| Cell type | Magnification | Resolution(μm/pixel) | Image Size | Image # (train/val/test) |
| --- | --- | --- | --- | --- |
| MDA-MB-231 | 20× | 0.621 | 512×512 | 216/56/56 |
|  | 40× | 0.311 | 512×512 | 336/72/72 |
| iPSC | 40× | 0.311 | 512×512 | 32/16/16 |

**Table S3. The PCC value of the model combination strategy for F-actin. A weighted combination of U-Net and cGAN from 0.1:0.9 to 0.9:0.1 with an incremental step of 0.2 was explored.**

| **Cell Type** | **U-Net/cGAN combination ratio** | | | | | | |
| --- | --- | --- | --- | --- | --- | --- | --- |
|  | **0.0:1.0** | **0.1:0.9** | **0.3:0.7** | **0.5:0.5** | **0.7:0.3** | **0.9:0.1** | **1.0:0.0** |
| 231 20× | 0.6991 | 0.7138 | 0.7180 | 0.7209 | 0.7197 | 0.7034 | 0.6723 |
| 231 40× | 0.7248 | 0.7327 | 0.7372 | 0.7287 | 0.705 | 0.6651 | 0.6492 |
| IPSC× | 0.6594 | 0.6682 | 0.6737 | 0.6775 | 0.6761 | 0.6637 | 0.6619 |

**Table S4. The PCC value of the model combination strategy for nuclei. A weighted combination of U-Net and cGAN from 0.1:0.9 to 0.9:0.1 with an incremental step of 0.2 was explored.**

| **Cell Type** | **U-Net/cGAN combination ratio** | | | | | | |
| --- | --- | --- | --- | --- | --- | --- | --- |
|  | **0.0:1.0** | **0.1:0.9** | **0.3:0.7** | **0.5:0.5** | **0.7:0.3** | **0.9:0.1** | **1.0:0.0** |
| 231 20× | 0.869 | 0.8771 | 0.8820 | 0.8835 | 0.8801 | 0.8699 | 0.8619 |
| 231 40× | 0.8710 | 0.8751 | 0.8793 | 0.8771 | 0.8674 | 0.8490 | 0.8354 |
| IPSC× | 0.8338 | 0.8379 | 0.8402 | 0.8413 | 0.8357 | 0.8240 | 0.8363 |

**Table S5. Implementation details of the pretrain model and the train-from-scratch model.**

| Models | Optimizer | Initial Learning rate | Decay strategy | Max epoch | Batch size | Loss |
| --- | --- | --- | --- | --- | --- | --- |
| U-Net | Adam | 0.001 | Lambda LR | 150 | 2 | MSE |
| cGAN | Adam | 0.001 | Lambda LR | 150 | 2 | smoothed L1 |

**Table S6. Implementation details of the fine-tuning.**

| Models | Optimizer | Initial Learning rate | Decay strategy | Max epoch | Batch size | Loss |
| --- | --- | --- | --- | --- | --- | --- |
| U-Net | Adam | 0.0001 | Lambda LR | 80 | 2 | MSE |
| cGAN | Adam | 0.001 | Lambda LR | 80 | 2 | smoothed L1 |


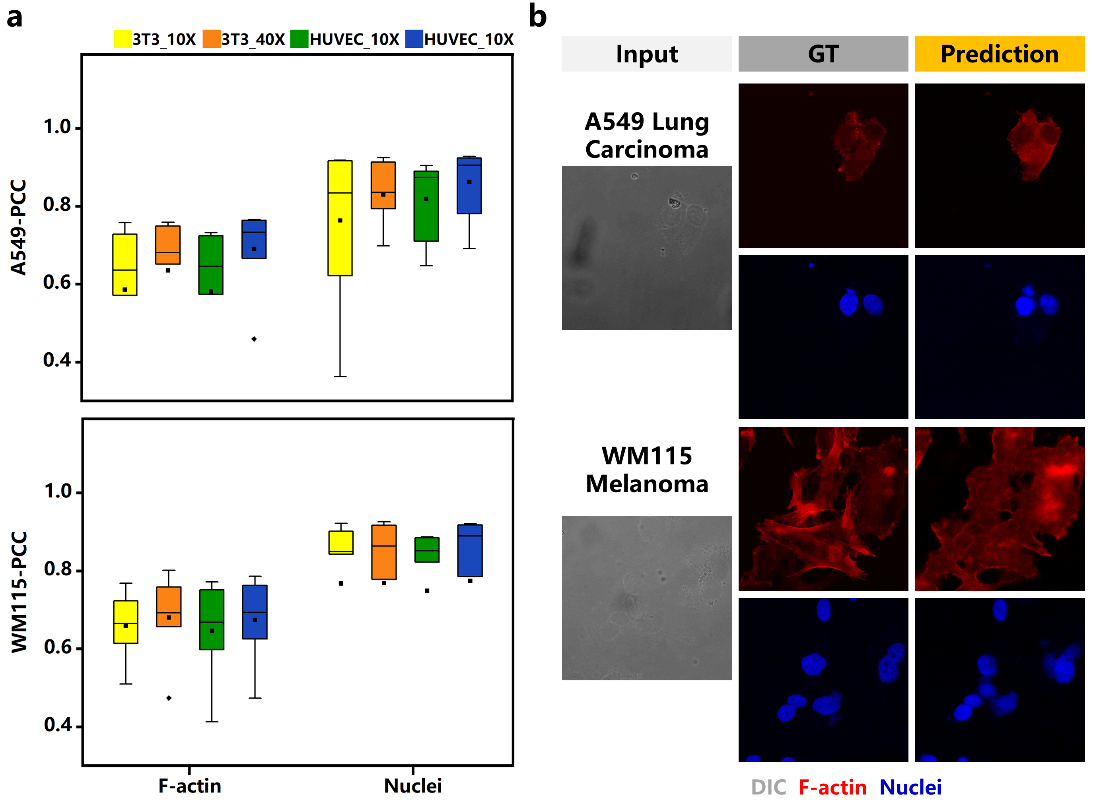


**Fig. S1. Generalizing CellVisioner to lung carcinoma cell images (A549) and melanoma cell images (WM115).** (a) PCC on the testing dataset. The matched datasets for A549 and WM115 were HUVEC 40× (blue box) and 3T3 40× (orange box). (b) Visualized virtual staining of F-actin and nuclei for A549 and WM115, respectively.


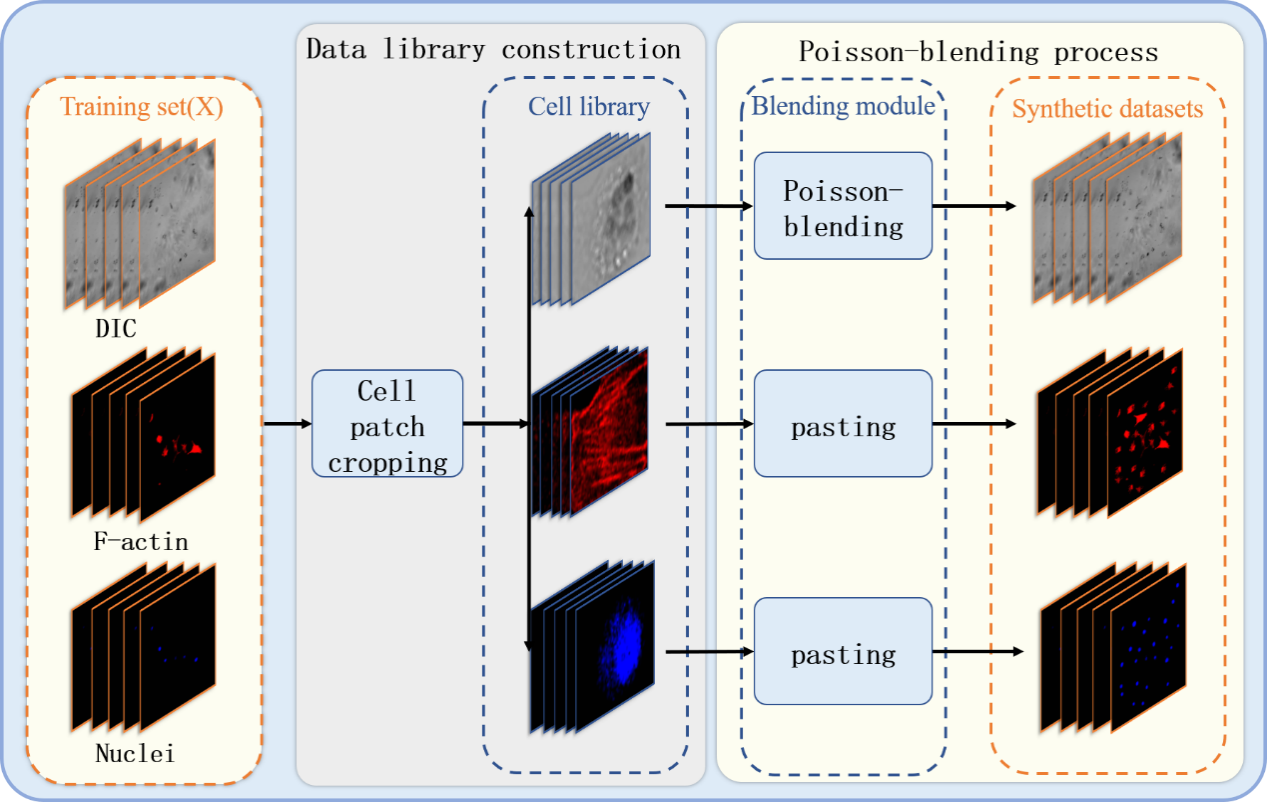


**Fig. S2.** **The flowchart of Poisson-blending data augmentation.** Single-cell image blocks are cropped from the training set to make up the cell patch library. DIC images are fused to the background image using the Poisson-blending algorithm. The corresponding fluorescence images of single-cells are directly pasted to the background fluorescence image.


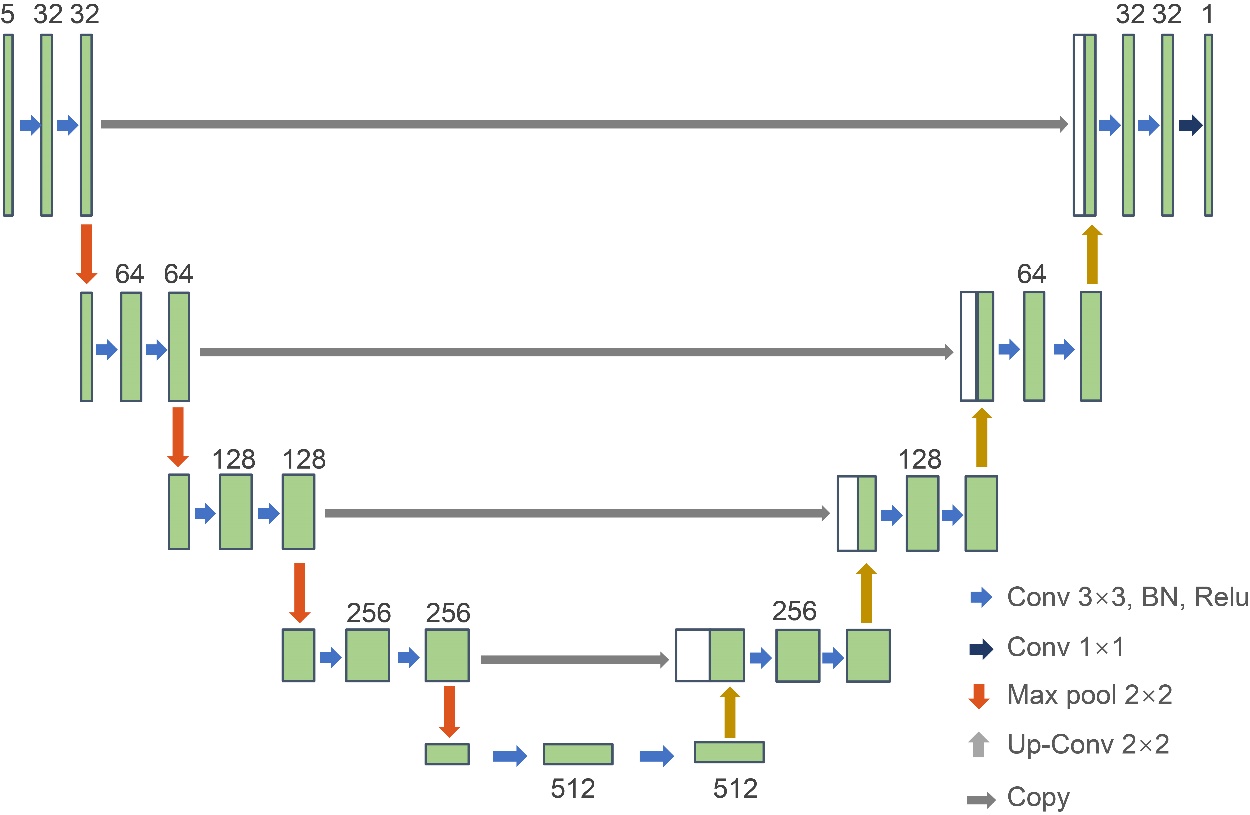


**Fig. S3. The U-Net architecture.** The U-Net architecture consists of a contracting path (left side) and an expansive path (right side).


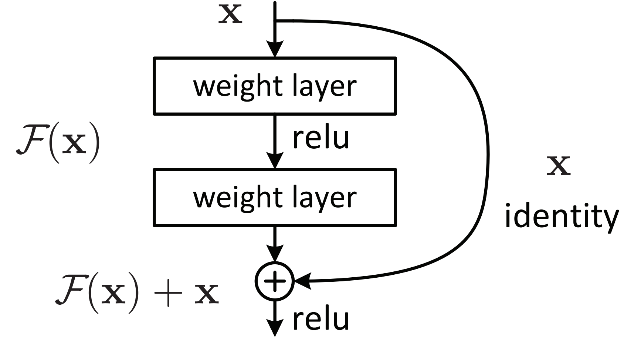


**Fig. S4. The residual unit in Res-UNet architecture [2].** Residual unit contains two 3×3 convolutional layers and a skip connection.


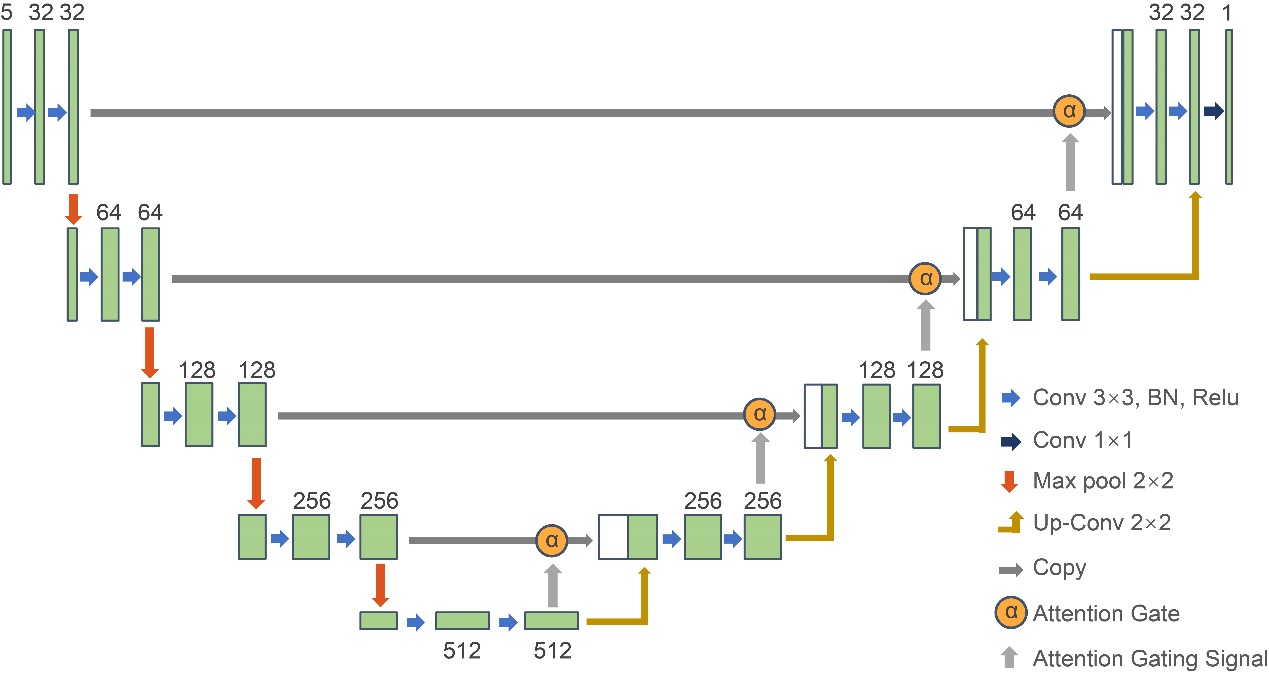


**Fig. S5. The att-UNet architecture.** Four attention gates are used to extend the standard U-Net model to generate better performance.


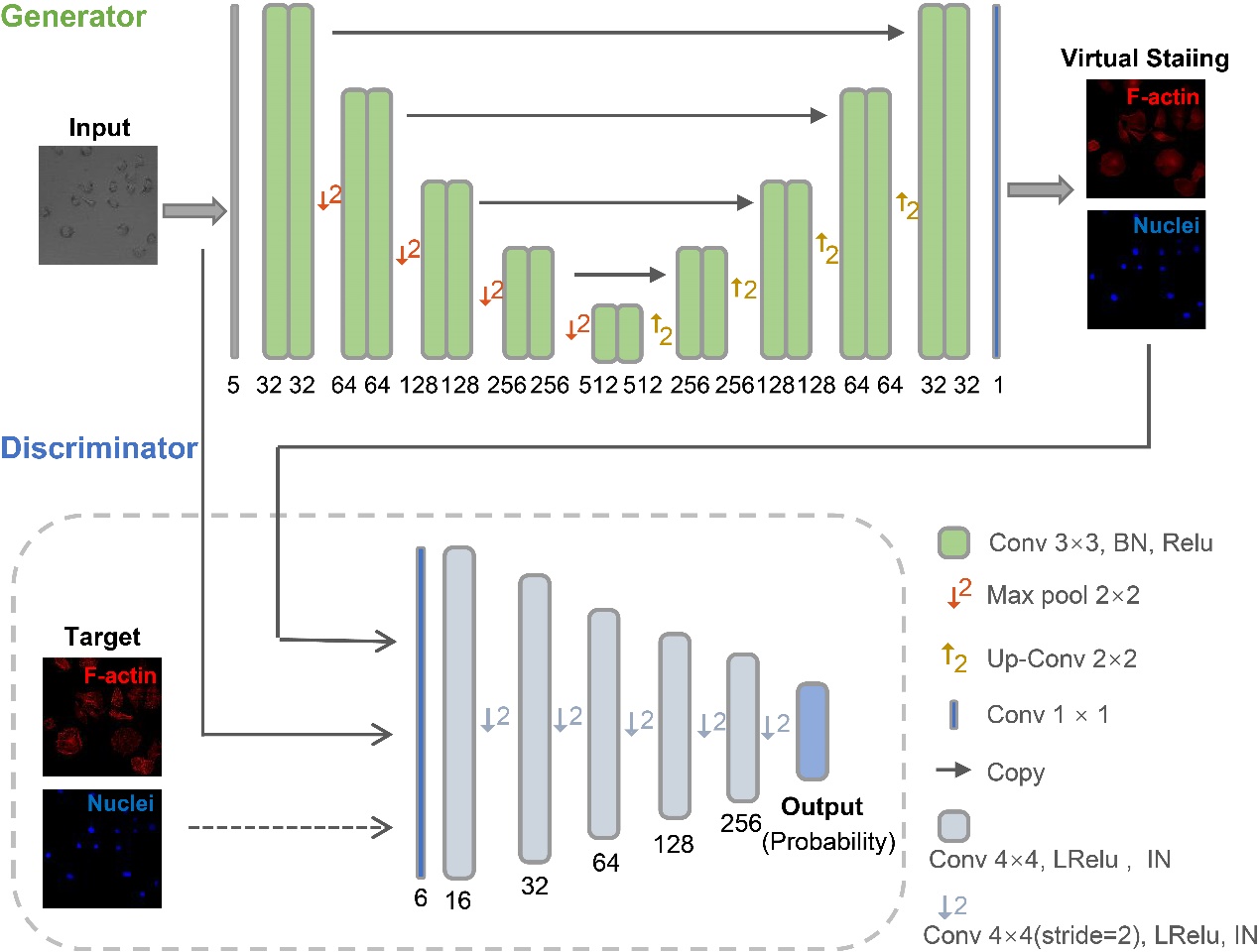


**Fig. S6. The cGAN architecture.** The generator network is U-Net model. The generator outputs an image and the discriminator learn to determine if the image is real.
